# Selection Effects on the Positioning of Genes and Gene Structures from the Interplay of Replication and Transcription in Bacterial Genomes

**Published:** 2007-10-09

**Authors:** Kazuharu Arakawa, Masaru Tomita

**Affiliations:** Institute for Advanced Biosciences, Keio University, Fujisawa, Kanagawa 252-8520, Japan

**Keywords:** GC skew, DNA replication, replicational selection, genome organization

## Abstract

Bacterial chromosomes are partly shaped by the functional requirements for efficient replication, which lead to strand bias as commonly characterized by the excess of guanines over cytosines in the leading strand. Gene structures are also highly organized within bacterial genomes as a result of such functional constraints, displaying characteristic positioning and structuring along the genome. Here we analyze the gene structures in completely sequenced bacterial chromosomes to observe the positional constraints on gene orientation, length, and codon usage with regard to the positions of replication origin and terminus. Selection on these gene features is different in regions surrounding the terminus of replication from the rest of the genome, but the selection could be either positive or negative depending on the species, and these positional effects are partly attributed to the A-T enrichment near the terminus. Characteristic gene structuring relative to the position of replication origin and terminus is commonly observed among most bacterial species with circular chromosomes, and therefore we argue that the highly organized gene positioning as well as the strand bias should be considered for genomics studies of bacteria.

## Introduction

Replication of bacteria with circular chromosomes start from a well-defined origin (*ori*), and the replication forks progress bidirectionally on the two replichores until both forks meet at the replication terminus (*ter*), usually located directly opposite of *ori* to maintain a physical balance (Rocha, 2004a; Rocha, 2004b). The asymmetrical replication machinery between that of the leading strand and that of the discontinuous replication in the lagging strand results in different mutational biases (Frank and Lobry, 1999; Lobry and Sueoka, 2002). This strand compositional asymmetry is known as the GC skew from the computational study of complete genome sequences, which shows an excess of G over C in the leading strand that abruptly shifts its polarity at *ori* and *ter* when this compositional bias is plotted along one strand of the chromosomal sequence (Arakawa et al. 2007a; Lobry 1996; Lobry and Louarn, 2003). The degree of GC skew generally correlates with the strength of replicational selection; for example, GC skew is either not evident or only weakly represented in intracellular parasites and in Cyanobacteria, which have a long doubling time in the order of hours to days (Kowalczuk et al. 2001; Salzberg et al. 1998; Worning et al. 2006). Fast growing bacterial species have minimal doubling time of less than one hour, and the functional requirement to conduct a highly efficient replication process exerts notable selectional pressures on the chromosomal organization of bacterial genomes, in addition to this compositional asymmetry. These functional constraints affect the orientation of several over-represented signal oligonucleotides, including the RAG motif recognized by the FtsK translocase, to locate the *dif* site in the *ter* region for the chromosomal dimer resolution by XerCD recombinase (Hendrickson and Lawrence, 2006; Levy et al. 2005; Pease et al. 2005; Perals et al. 2001; Perals et al. 2000), and the Chi sequence recognized by the RecBCD exonuclease/helicase for the recombinational repair of stalled replication forks (Arakawa et al. 2007b; Kowalczykowski et al. 1994; Uno et al. 2000; Uno et al. 2006).

Replicational selection also controls the organization and structures of genes. Genes are preferentially co-oriented in the direction of replication (i.e. preferentially located in the leading strand; 55% in *Escherichia coli*, 54% in *Haemophilus influenzae*, and 74% in *Bacillus subtilis*) (McLean et al. 1998), especially for essential genes (Rocha and Danchin, 2003), and transcription units and operons tend to be longer in length when located in the leading strand (Omont and Kepes, 2004; Price et al. 2005). These coding preferences are partly attributed to the head-on collision of DNA and RNA polymerases when the replication fork reaches a highly transcribed gene in the lagging strand, which either slows the movement of the replication fork or interrupts the transcription of essential genes (Brewer, 1988; Liu and Alberts, 1995; Price et al. 2005). Fast growing bacteria often have multiple rounds of replication to compensate the limitation in the maximal rate of the DNA polymerases, and since this results in dosage effects near the origin of replication, transcription and translation genes are preferentially located near *ori*, in the leading strand (Couturier and Rocha, 2006). Coupled with the G + C composition at the third codon position (GC3), the codon usage of genes is also reported to be affected by the polarity of genomic composition, especially by the A + T enrichment near *ter* (Daubin and Perriere, 2003).

In this work, we analyzed the genomes of 300 bacterial species with circular chromosomes for the genomic organization of gene length, orientation, and codon usage bias relative to the positions of *ori* and *ter*, in order to understand the effects of gene positioning within the genome. As a result, this selection on the gene structures was commonly observed in most bacterial species, displaying characteristic organizations that are different in regions surrounding *ter* but not necessarily proportional to the distance from *ter*. We therefore suggest that the genomic studies of the gene structures should consider the background effects of the genomic positions.

## Methods

### Sequences and software

Complete genome sequences in GenBank format were obtained from the NCBI RefSeq FTP repository (ftp://ftp.ncbi.nih.gov/genomes/Bacteria/). In this study we have used 300 complete genome sequences of eubacteria that have circular chromosomes. All analyses were conducted using the G-language Genome Analysis Environment version 1.6.4 (Arakawa et al. 2003; Arakawa and Tomita, 2006). The positional coordinate system for the genomic sequence used in this work was set to originate at 0, unlike that of GenBank, which uses 1 for the origin. Leading and lagging strands were defined from the locations of *ori* and *ter*, predicted using the maxima and minima of cumulative GC skew graphs with single base pair resolution (Frank and Lobry, 2000), or documented with experimental confirmation where available (Freeman et al. 1998): *E. coli* K12 MG1655 [GenBank: NC_000913] at 3923499 and 1588799, *H. influenzae* [GenBank:NC_000907] at 602999 and 1517999, and *B. subtilis* [GenBank:NC_000964] at 0 and 2016999, for *ori* and *ter* respectively.

### Analysis of gene structures

Gene structures are controlled by various functional requirements, and therefore contain considerable number of constraints in addition to the replicational selection, which results in signal “noise” when observed relative to the distance from the origin. To level out local noise and to make the positional tendencies apparent, cumulative diagrams were utilized (Grigoriev, 1998). Gene orientation was given a score of the ratio between the fraction of genes in the lagging strand over that in the leading strand (i.e. score < 1) when the gene was in the leading strand, and −1 when in the lagging strand, in order to normalize the score to account for the different frequencies of genes in the two strands, and to enable effective cumulative plotting. Similarly, gene length was given a score of 1 when the length was longer than mean, and −1 when shorter. For codon bias measures, cumulative sum of Codon Adaptation Index (CAI) (Sharp and Li, 1987) centered on the mean was calculated by subtracting the mean CAI value for all genes from the observed CAI value, so that the value is positive when greater than mean and negative when below (Daubin and Perriere, 2003), using the reference set of ribosomal genes. CAI measures the relative adaptiveness of the codon usage of a gene towards the reference set, and is mostly utilized to estimate the expressiveness of genes by using highly expressed genes such as the ribosomal genes as the reference set, and structuring of CAI is reported to be similar to other codon bias measures (Daubin and Perriere, 2003). Ribosomal proteins (only 71 genes in *E. coli*) were excluded for the cumulative plot of CAI, since they are used as the reference set and therefore results in extremely high CAI values, and since they tend to be clustered within the genome and produce sharp local peaks that deform the cumulative graphs.

### Verification of the contribution of gene essentiality to strand bias

Gene essentiality is reported to be a major driving force for the strand bias of gene positioning in *E. coli* (Rocha and Danchin, 2003), and in order to test this contribution of essentiality on the four features of gene structure analyzed in this work, we have repeated the same analyses after removing the essential genes. Information about gene essentiality was obtained from the Profiling of *E. coli* Chromosome (PEC; http://www.shigen.nig.ac.jp/ecoli/pec/) database, where a total of 250 essential genes including 234 essential protein-coding genes are listed based on comprehensive gene disruptions (Hashimoto et al. 2005).

### Comparison with the degree of skew

To compare the results of the gene structure with the strength of mutational selection in leading and lagging strands, GC Skew Index (GCSI) was calculated for all the available complete circular bacterial chromosomal sequences. GCSI quantifies the strength of GC skew using the power spectrum of Fourier transform of the GC skew graph and the Euclidean distance between the peaks (Arakawa and Tomita, 2007).

### Statistical assessment of the positional effects near *ter*

To test whether the distribution of the gene structures are different around the regions surrounding *ori* and *ter*, cumulative values were calculated with genes ordered according to their distances from *ori*. Highest peak, either maximum or minimum depending on the greatest absolute distance from the baseline, was used as the parting position to divide the genome into two regions, one surrounding *ori* and the other surrounding *ter*. Distribution of the gene structures in these two regions were tested using the distribution of original values (as opposed to the cumulative value) with two-sided Welch two sample t-test, because equal distribution in these two regions could not be assumed. Difference of the distribution in the two regions were noted using three confidence levels according to the p-values: * for p < 0.01, ** for p < 0.001, *** for p < 0.0001.

## Results and Discussion

Here we have analyzed the positional effects on the gene structure of bacterial chromosomes with regard to the distance of genes from the replication origin and terminus, in order to understand the replicational selection effects on the strand bias of gene structures. Cumulative graphs were utilized with statistical assessment for this purpose. Gene structural features, including the orientation, length, and expressiveness or codon usage, have been reported to be positively selected in the leading strand in several previous works (McLean et al. 1998; Omont and Kepes, 2004; Price et al. 2005), and A-T enrichment near *ter* correlating with the cumulative CAI values was pointed out (Daubin and Perriere, 2003), but their comprehensive distribution pattern in the genome, which is the focus of this work, had not been reported.

**Figure 1.**
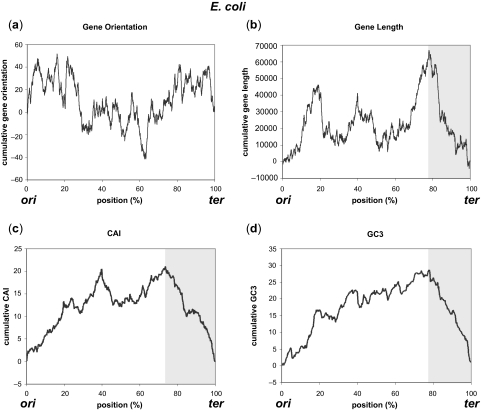
Strand bias features of gene structures cumulatively plotted for *E. coli*. (**a**) Gene orientation, (**b**) Gene length, (**c**) CAI, and (**d**) GC3. The shaded area represents regions around *ter* where the distribution is shown to be significantly different from that of the rest of the genome.

**Figure 2.**
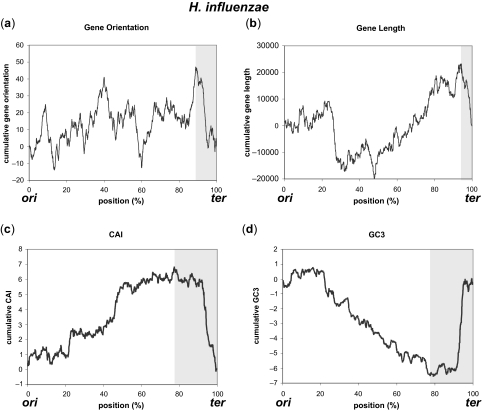
Strand bias features of gene structures cumulatively plotted for *H. influenzae*. (**a**) Gene orientation, (**b**) Gene length, (**c**) CAI, and (**d**) GC3. The shaded area represents regions around *ter* where the distribution is shown to be significantly different from that of the rest of the genome.

**Figure 3.**
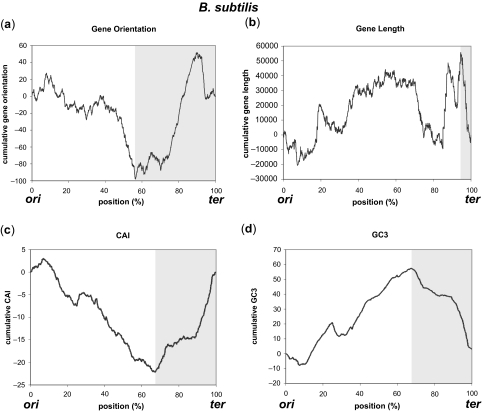
Strand bias features of gene structures cumulatively plotted for *B. subtilis.* (**a**) Gene orientation, (**b**) Gene length, (**c**) CAI, and (**d**) GC3. The shaded area represents regions around *ter* where the distribution is shown to be significantly different from that of the rest of the genome.

**Figure 4.**
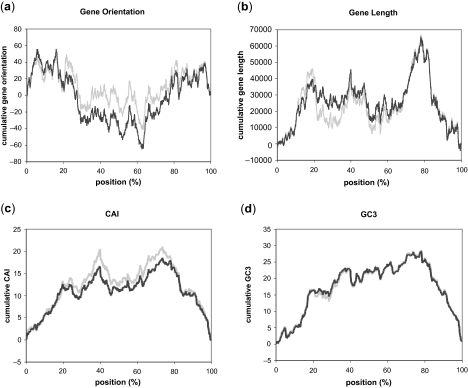
Strand bias features of gene structures in *E. coli*, after removal of essential genes. (**a**) Gene orientation, (**b**) Gene length, (**c**) CAI, and (**d**) GC3. Dark lines show the graphs after removal, and the gray lines show those before removal.

Strand bias features were plotted as cumulative graphs originating from *ori* to *ter* to show the positional effects on gene structures. Graphs are shown for three species (Figs. 1–3, for *E. coli*, *H.influenzae*, and *B.subtilis*, respectively), in which the locations of *ori* and *ter* are experimentally identified. Statistical information for 50 genomes out of 300 genomes (first 50 ordered by their accession numbers) analyzed in this work is depicted in Table 1 (See Supplementary Information for comprehensive listing of the results for all 300 genomes). Except for gene orientation in *E. coli* that did not show statistically significant positioning, all of the other gene structures in the three genomes exhibited different distribution around *ter* compared with the distribution of the rest of the genome. No correlation between the gene structure and the distance from *ori* or *ter* was observed. These characteristic changes in distribution were observed throughout many bacterial species, most being highly significant (marked *** for p < 0.0001). Gene essentiality is a major driving force of gene positioning and strand selection (Rocha and Danchin, 2003), but the removal of essential genes in *E. coli* had little effect on the overall shape of the cumulative graph, and since the majority of essential genes were located close to *ori*, the identified region surrounding *ter* was not affected by this removal (Fig. 4).

In species where the distribution of certain gene structure was significantly different from that of the rest of the genome (p < 0.01), genes were more oriented in the direction of replication near *ter* for 168 out of 255 (65.9%) genomes, shorter near *ter* for 91 out of 161 (56.5%), CAI was below average for 147 out of 265 (55.5%), and enriched in A-T for 212 out of 271 (78.2%). Therefore, most genomes showed A-T enrichment near *ter*, but the characteristic distribution near *ter* was different among the analyzed genomes. For example, *E. coli* genome showed descending graphs in the significant region near *ter* (Fig. 1 b–d), but the region was enriched in G-C in *H. influenzae* (ascending, Fig. 2 d) even though other gene structures showed descending graphs (Fig. 2 a–c). Likewise, the graphs of CAI and GC3 were inversely shaped in *B. subtilis* (Fig. 3 c–d), as indicated in previous studies (Daubin and Perriere, 2003). Nevertheless, most genomes showed different distribution patterns for the gene structures in the proximity of *ter*, mostly encompassing 5 to 40% of the genome. As indicated by the low p-values, distribution of gene structures in these regions surrounding *ter* was significantly different from those of the whole genome. For example, average gene length in *E. coli* is 951.87 bp, but that around *ter* is 884.16 bp (972.50 bp for the rest of the genome). Average gene length in *H. influenzae* is 937.75 bp for whole genome and 741.15 bp around *ter* (952.97 bp for the rest of the genome), and that in *B. subtilis* is 895.63 bp for whole genome and 687.86 bp around *ter* (910.20 bp for the rest of the genome). Similarly, gene orientation rate for the direction of replication in *H. influenzae* is 54.32% for whole genome and 30.72% around *ter* (57.55% for the rest of the genome), and that in *B. subtilis* is 73.67% for whole genome and 78.99% around *ter* (69.31% for the rest of the genome). Significant regions around *ter* were very limited in size (around 5%) for gene length in *H. influenzae* and *B. subtilis*, but even these limited regions lowered the average value for the rest of the genome (occupying 95% of the genome). Therefore, genomic studies of the gene structures should carefully consider the positioning effects of genes on their properties especially around *ter*, and general observations using the entire genome may not be always suitable, considering these biased distributions.

Only the peak position of CAI and GC3 showed notable correlation: *R^2^* values were 0.1376 for species scoring p < 0.01 and 0.2728 for p < 0.001, and strong correlation of *R^2^* = 0.6567 was observed for p < 0.0001. Lack of correlation suggests that the positional effects on gene length and orientation are independent of each other, and they are not the results of local GC content. Conversely, the strong correlation of the peak positions of CAI and GC3 in most bacterial species indicates that the two properties are related. Change in the local GC content affects the frequencies of codons, and this is presumably the main cause for this correlation, as discussed in previous studies (Hooper and Berg, 2003). Inverse correlation of CAI and GC3 in *B. subtilis* is also indicative of this effect of local GC content on CAI, since major codons in *B. subtilis* tend to have low GC3 (Sharp et al. 1988). Expressiveness of genes should be further investigated if quantitative expression data becomes available for a large number of species, in order to validate whether the local GC content drives the mutational bias or the positioning of highly expressed genes drives the strand specific mutational bias.

Correlation of the peak position and GCSI values was not observed for any of the properties of the genes. GC skew is not evident in slow growing bacteria such as Cyanobacteria (Salzberg et al. 1998), and replicational selection is partly attributed to the mutational bias in the leading and lagging strands (Kowalczuk et al. 2001). Differing distribution patterns near *ter* for the enrichment of AT and subsequently the corresponding change in CAI, as well as the changes in gene orientation percentage and average gene length, were observed commonly among most bacterial species analyzed in this work, indicating replicational selection on the gene structures. Selectional pressure of the replicational machinery around *ter* is presumably lower than around *ori*, and the peak position is indicative of the boundary up to which the replication selection is strong. GC skew results from a kind of strand-specific mutational bias and is compatible in most cases with a neutral and purely mutational hypothesis (Lobry and Sueoka, 2002), therefore the replicational selection acting on the four gene structural properties analyzed probably involve complex interplay of replication and transcription other than the mutational bias.

The gene structures analyzed in this work are constrained by the selection exerted by the replication machinery, and thus are distributed differently around the regions surrounding *ter*. The reason for this selection requires further investigation, but possible explanations are suggested, for example, by the reduced requirements for efficient replication around *ter* compared to *ori*, to avoid head-on collisions of DNA and RNA polymerases (Omont and Kepes, 2004), to ascertain effective expression (Price et al. 2005), and to make use of the dosage effects (Couturier and Rocha, 2006). Our results imply the importance of considering the highly selected region near *ter* in addition to the differences in the leading and lagging strands, since replicational selection does not necessarily affect the entire leading strand, and the effect may be different near *ter*. These positional effects within the genome should provide deeper insights into the structuring of the bacterial genome through replicational selection in evolution.

## Supplement Materials
